# The adaptability of soybean photosynthesis to midday high-light duration through CEF-NPQ coupling regulation

**DOI:** 10.3389/fpls.2025.1648079

**Published:** 2025-09-09

**Authors:** Yi Lei, Xiaoling Wu, Jing Gao, Qi Wang, Jingru Wang, Dhungana Diwakar, Xianming Tan, Feng Yang, Wenyu Yang

**Affiliations:** ^1^ College of Agronomy, Sichuan Agricultural University, Wenjiang, Chengdu, China; ^2^ Sichuan Engineering Research Center for Crop Strip Intercropping System ,Sichuan Agricultural University, Chengdu, China; ^3^ Key Laboratory of Crop Ecophysiology and Farming System in Southwest, Ministry of Agriculture and Rural Affairs, Chengdu, China

**Keywords:** intercropping, photosynthesis, photoprotection, cyclic electron flow, light density

## Abstract

Fluctuating light (FL) conditions particularly the diurnal alternation between shaded and high-light periods are intrinsic to intercropping systems and impose substantial regulatory challenges on crop photosynthesis. However, the cultivar-specific mechanisms underlying adaptation to such dynamic light environments remain largely unexplored. Here, we examined how the duration of midday high-light exposure modulates the coordination between cyclic electron flow (CEF) and non-photochemical quenching (NPQ) in two soybean cultivars grown under simulated intercropping light regimes. Plants were exposed to morning shade followed by either short (T30) or prolonged (T150, T200) midday high-light treatments. All treatments triggered common photoprotective responses, including increased energy dissipation (DIo/CSm, +18.7–22.3%) and reduced electron transport efficiency (ETo/CSm, −14.2–17.5%). Yet, the cultivars exhibited distinct photoregulatory strategies depending on light duration. The light-adapted cultivar ND12 rapidly established a proton gradient (ΔpH; 34.8% faster) and sustained higher PSII efficiency (ETRII, +41.5%) under brief high-light exposure, indicating a preemptive ΔpH priming mechanism. In contrast, the light-sensitive GX7 required extended high-light duration (T200) to induce CEF (+60.5%) and plastoquinone pool expansion (+22.0%), suggesting a delayed, duration-dependent adjustment strategy. These cultivar-specific responses ultimately enhanced photosynthetic performance by 34.8–52.4% under FL conditions. Our findings offer mechanistic insights into how midday light duration shapes genotype-dependent photosynthetic regulation, providing a physiological basis for optimizing light utilization in intercropping systems.

## 1 Introduction

Dynamic light environments profoundly shape crop photosynthesis, growth, and productivity. In natural field conditions, diurnal solar angle changes generate a characteristic triphasic irradiance pattern (“low–high–low”), with pronounced midday peaks (Lazzarin et al., 2024). However, in intercropping systems—such as maize–soybean—this pattern is further modulated by canopy structure (Yang et al., 2015; Li et al., 2023). Spatial niche differentiation and row configurations create alternating weak–strong light environments within the understory, especially during midday, where understory crops experience fluctuating exposure to high irradiance (Raza et al., 2020; Feng et al., 2024). These structural light transitions, rather than simple temporal peaks, present a complex and underexplored challenge to photosynthetic regulation.

While light is essential for photosynthesis, both insufficient and excessive irradiance pose constraints on crop productivity. Understory crops often experience suboptimal light before abruptly encountering intense irradiance at midday—conditions that can induce photoinhibition, particularly when light levels exceed 1200 µmol m^−2^ s^−1^ (Yamori, 2016). Current understanding of photosynthetic light responses largely stems from constant irradiance studies (Dang et al., 2023; Qiang et al., 2024), which do not capture the rapid transitions and spatial heterogeneity characteristic of intercropped field environments. In multi-row relay systems, midday light penetration may increase 4–5-fold depending on cultivar and canopy structure (Fan et al., 2019; Li et al., 2023). underscoring the need for mechanistic insights into photosynthetic acclimation under such fluctuating light.

Plants respond to fluctuating light within seconds to minutes by dynamically adjusting biochemical and biophysical processes that regulate light capture and energy dissipation (Tanaka et al., 2019; Wang et al., 2025). Photosynthetic electron transport generates a proton motive force (pmf), comprising a proton gradient (ΔpH) and membrane potential (ΔΨ), which coordinates energy conversion and protective responses (Kramer et al., 2003). Cyclic electron flow (CEF) around PSI is a key modulator of pmf, promoting ΔpH buildup without net NADPH production—thereby balancing ATP demand (Wang et al., 2022a; Chauhan et al., 2023) and activating non-photochemical quenching (NPQ) (Goss and Lepetit, 2015; Slattery et al., 2018). This feedback system is critical during low-to-high light transitions, where rapid lumen acidification protects PSII and prevents photoinhibition (Armbruster et al., 2017).

Genotypic variation significantly influences how quickly and efficiently crops acclimate to fluctuating light. In wild-type angiosperms, photoinhibition of PSI often occurs within the first 20 seconds of high-light exposure due to delays in ΔpH establishment (Alter et al., 2012; Sakoda et al., 2016; Ferroni et al., 2020). Moreover, while NPQ induction is rapid, its relaxation during HL-to-LL transitions is much slower, creating mismatches in light energy use efficiency (Slattery et al., 2018; Goss and Lepetit, 2015). Optimizing both the induction and relaxation kinetics of CEF and NPQ is therefore vital to improving light-use efficiency under naturally fluctuating or structurally induced light regimes.

Despite the recognized role of photosynthetically active radiation (PAR) in plant growth, the mechanistic basis by which soybean cultivars regulate electron transport and photoprotective responses under varying midday light durations remains poorly understood. Soybean(*Glycine max (L.) Merr.*), a facultative short-day crop, is particularly sensitive to light quality and quantity variations. Particularly, how these cultivars coordinate CEF–NPQ responses during transitions from low to high light defining feature of intercropped canopies—has not been systematically studied. To address this gap, we measured natural light patterns in the field and simulated varying midday high-light durations under controlled conditions. We reveal cultivar-specific strategies in photosystem regulation, ΔpH priming, and photoprotective activation, offering novel insights into optimizing photosynthetic performance in intercropping systems.

## 2 Materials and methods

### 2.1 Plant material and experimental design

**Figure 1:**
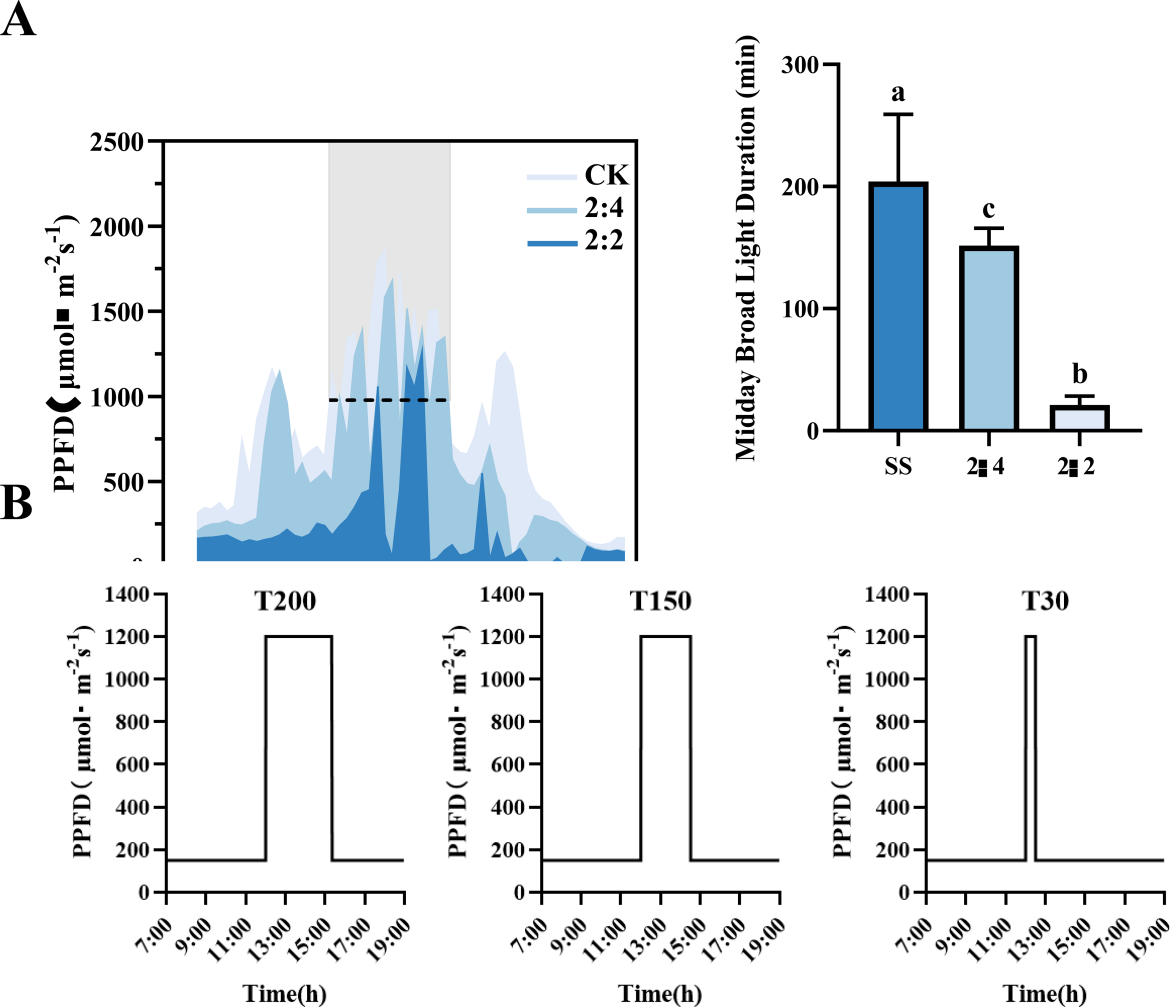
Field light environment and climate light environment simulation. **(A)** Diurnal variations of the photosynthetic photon flux density (PPFD) in the soybean layer under different maize-soybean intercropping configurations (2:2, 2:3, 2:4). **(B)** Experimental light treatments (T30, T150, T200) simulating different configurations. Different letters denote significant differences (P < 0.05) among treatments.

Two soybean (Glycine max L.) cultivars with contrasting light response characteristics were used. ND12 is a light-adaptive genotype developed by the Nanchong Academy of Agricultural Sciences (Sichuan, China), characterized by strong photoprotective capacity and rapid response to dynamic light. GX7, developed by the Guangxi Academy of Agricultural Sciences (Guangxi, China), is a light-sensitive genotype that tends to exhibit delayed photosynthetic adjustment under fluctuating light (Gao et al., 2024). Seeds were germinated in a 1:3 (v/v) sterilized mixture of vermiculite and nutrient soil (Danish Pinstrup Substrate) pre-treated with carbendazim. Plants were grown under controlled conditions at Sichuan Agricultural University (Chengdu, China), with a 12 h light/12 h dark photoperiod, daytime temperature of 25 °C, nighttime 22 °C, and relative humidity of 60–70%. To establish environmentally relevant treatments, we first conducted field measurements of photosynthetically active radiation (PAR) using an LI-191R line quantum sensor (LI-COR, USA). Measurements were made under clear-sky conditions at 1-meter height within the soybean canopy from 09:00 to 18:00, at one-minute intervals across different maize–soybean relay intercropping row configurations. The baseline low-light intensity of 150 μmol m^−2^ s^−1^ used outside the midday period was chosen to reflect field conditions observed during morning and late afternoon hours in the canopy. Based on our PAR measurements (**Figure 1A**), these periods consistently showed values ranging between 120 and 180 μmol m^−2^ s^−1^, making 150 μmol m^−2^ s^−1^ an appropriate representative intensity for simulating ambient light levels under relay intercropping. These assessments revealed that midday high-light duration (PPFD >1200 µmol m^−2^ s^−1^) increased significantly in widened row configurations: 400.00% in 2:3 and 566.67% in 2:4 compared to the 2:2 configuration, while maintaining optimal light quality and quantity (**Figure 1A**).

These field-derived light dynamics informed a simulation of three distinct midday high-light durations in a growth chamber using a programmable LED system: T30 (30 min at 1200 µmol m^−2^ s^−1^, simulating 2:2), T150 (150 min, simulating 2:3), and T200 (200 min, simulating 2:4). Outside of the midday period, a baseline low-light condition of 150 µmol m^−2^ s^−1^ was maintained. Treatments were applied beginning at 12:00 h, and plants were allowed to recover under low light afterward. Each treatment was performed in two independent experimental batches. In each batch, the treatment was replicated three times, with ten soybean plants per replicate (30 plants total per treatment per batch).

### 2.2 Measurement of the dynamic response of photosynthesis

The dynamic response of photosynthesis was assessed using a LI-6800 portable photosynthesis system (LI-COR, Inc., Lincoln, NE, USA) on intact soybean compound leaves. The photosynthetically active radiation (PAR) in the leaf chamber was set to 150 μmol·m^−2^ s^−1^·sm^−2^ s^−1^, and the CO_2_ concentration was maintained at 400 μmol·mol m^−1^. Soybean leaves were clamped into the chamber and acclimated for 20–30 minutes. Once photosynthetic parameters stabilized, the light intensity was increased to 1200 μmol·m^−2^·s^−1^ for 30 minutes before being reduced back to 150 μmol·m^−2^·s^−1^. The measurement results were fitted to calculate the response time of the net photosynthetic rate (Pn) to the light intensity during the transitions from low light to high light and from high light to low light. Prior to measurement, five morphologically uniform and healthy plants were selected from each treatment group within each batch. The selection was based on similar plant height, leaf area, and overall appearance, ensuring biological consistency. These five plants were used for all physiological and morphological measurements. The final results were obtained by averaging two independent batches of seedlings.

Calculating the time to 50% or 90% of the rate maximum for Pn, The induction kinetics for a given photosynthetic parameter can be expressed using an exponential model as follows (Mott and Woodrow, 2000):


$$F(t)=Fmax+(Fmin-Fmax)e^{-1/\tau -i\omega t}$$

where Fmin and Fmax represent the minimum and maximum of Pn during photosynthetic induction, and τ represents the time constants for that parameter.

### 2.3 Measurement of the kinetics of chlorophyll fluorescence induction

After a 30-minute dark acclimation period, the kinetics of chlorophyll fluorescence induction were measured using a Plant Efficiency Analyzer (HandyPEA, Norfolk, UK). Fluorescence transients were recorded over a 1-second light pulse, and the OJIP curve was analyzed using the JIP-test, as described in previous studies. Measurements were taken from the middle portion of a healthy compound leaf, with 10 plants sampled per treatment. The following fluorescence parameters were recorded: maximum fluorescence intensity (Fm), minimum fluorescence intensity at 20 μs (Fo), and fluorescence intensities at 2 ms (J-step) and 30 ms (I-step). The variable fluorescence yield (Fv), defined as Fm − Fo, was used to calculate the maximum quantum yield of photosystem II (PSII) as Fv/Fm = 1 − Fo/Fm. Additionally, when maximum fluorescence (Fm) was reached, the following parameters were calculated:

ABS/CSm – energy absorbed per unit leaf cross-sectional area (approximated by Fm).

TR_0_/CSm – energy captured per unit leaf cross-sectional area.

ET_0_/CSm – energy transferred through electron transport per unit leaf cross-sectional area.

DI_0_/CSm – energy dissipated as heat per unit leaf cross-sectional area

### 2.4 Measurement of photosystem parameters and photosynthetic electron transfer

Photosystem II (PSII) parameters were measured using a Dual-Channel Modulated Chlorophyll Fluorometer (DUAL-PAM-100, WALZ, Germany). A saturating pulse of 20,000 μmol·m^−2^·s^−1^ was applied for 300 milliseconds. After at least 20 minutes of dark acclimation, the redox state of the photosystem I (PSI) reaction center was analyzed by measuring the absorbance of P700 following a predefined protocol.

To determine the dark-reduction kinetics of P700, measurements were performed immediately after obtaining the dark-reduction curve, without re-acclimating the plants to darkness. The plastoquinone (PQ) pool size was then measured using a sequential light exposure protocol: the measurement protocol began with the activation of far-red light, followed by a 10-second waiting period to allow for initial stabilization. Next, a single-turnover saturating flash (ST) was applied for 10 seconds to induce a rapid electron transfer response. This was followed by the initiation of multiple-turnover saturating flashes (MT) for 30 seconds to further assess the plastoquinone (PQ) pool dynamics. Finally, the far-red light was deactivated to conclude the measurement. Following data collection, PSI and PSII parameters were calculated as **Supplementary Table 1** to the **Supplementary Information (SI)** section.

### 2.5 Analysis of the electrochromic shift

Using the Dual PAM-100 (Walz) equipped with a P515/535 emitter-detector module, the ECS signal was monitored as the absorbance change at 515 nm. After the plants were fully dark-adapted, the measurement was initiated. When the curve leveled off, the actinic light was turned on. After 600 seconds of illumination with the actinic light, it was turned off. At this time, the P515 value decreased rapidly and then increased. After the curve stabilized, the measurement program was terminated. From the dark-light-dark curve changes of the 550–515 nm signal, two components of the transmembrane proton motive force (pmf) could be obtained: the proton gradient (ΔpH) and the membrane potential (ΔΨ). All ΔpH and ΔΨ levels were normalized according to the amplitude of the ECS and calculated using the DIRK analysis.

### 2.6 Dynamic responses of the photosystem to alternating low and high intensity

After the plants were fully dark-adapted, the rapid responses of the PSI and PSII parameters were measured at 25°C using the Dual-PAM 100 measurement system (Heinz Walz, Effeltrich, Germany). The leaves were illuminated with 150 μmol·m^−2^·s^−1^ for 40 seconds, followed by illumination with 1200 μmol·m^−2^·s^−1^ for 80 seconds, and then with 150 μmol·m^−2^·s^−1^ for 80 seconds. During the alternation of low-intensity, high-intensity, and low-intensity light, the PSI and PSII parameters were measured (the formulas are consistent with those in section 2.5).

### 2.7 Statistical analysis

The results are presented as mean values obtained from five biological replicates per treatment per batch (n = 5), averaged across two independent experimental batches (total n = 10). Statistical significance was determined using LSD tests (p < 0.05) in SPSS 26.0. GraphPad Prism8.0 was used for graph plotting.

## 3 Results

### 3.1 Effects of midday high-light duration on soybean growth

**Figure 2:**
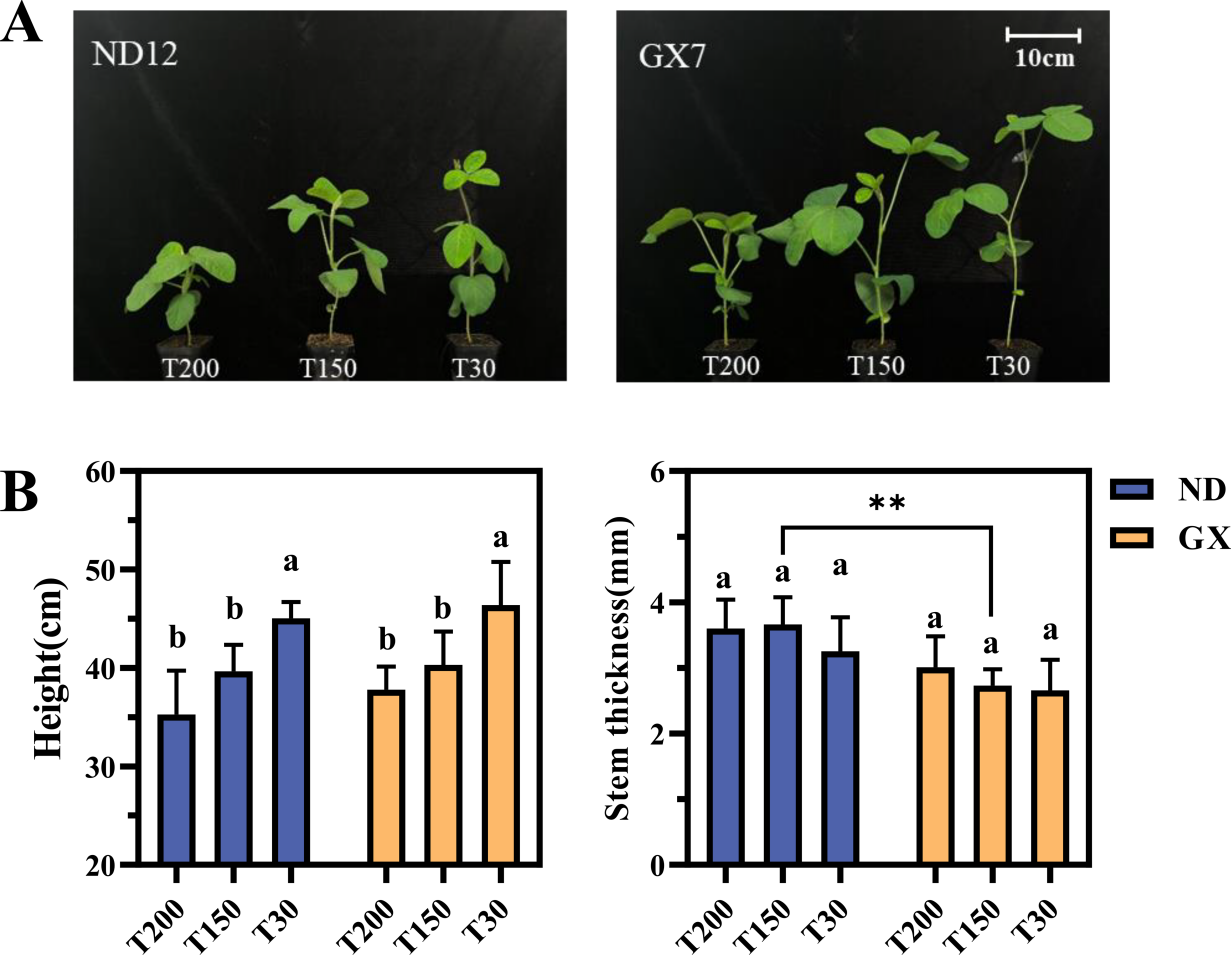
Effects of Simulated Light on Soybean Growth. **(A)** Effect of light treatments on the growth of soybean. **(B)** Effects of treatments with different durations of intense midday light on the plant height and stem diameter of soybeans. Plant height was measured from the soil surface to the shoot apical meristem and the scale bar applies to both images. ** denotes highly significant ones (p < 0.01).

The impact of these light regimes on morphological traits was first evaluated (**Figures 2A, B**). Both cultivars showed significant reductions in plant height under prolonged midday high light, with ND12 decreasing by 47.52% and 81.46% under T200 compared to T150 and T30, respectively, and GX7 showing even greater reductions of 75.62% and 96.90%. Conversely, stem diameter increased in T200: ND12 showed increases of 6.16% and 14.48% over T150 and T30, while GX7 increased by 26.32% and 15.60%.

### 3.2 Effects of midday high-light duration on photosynthetic performance and light energy utilization efficiency in soybean

**Figure 3:**
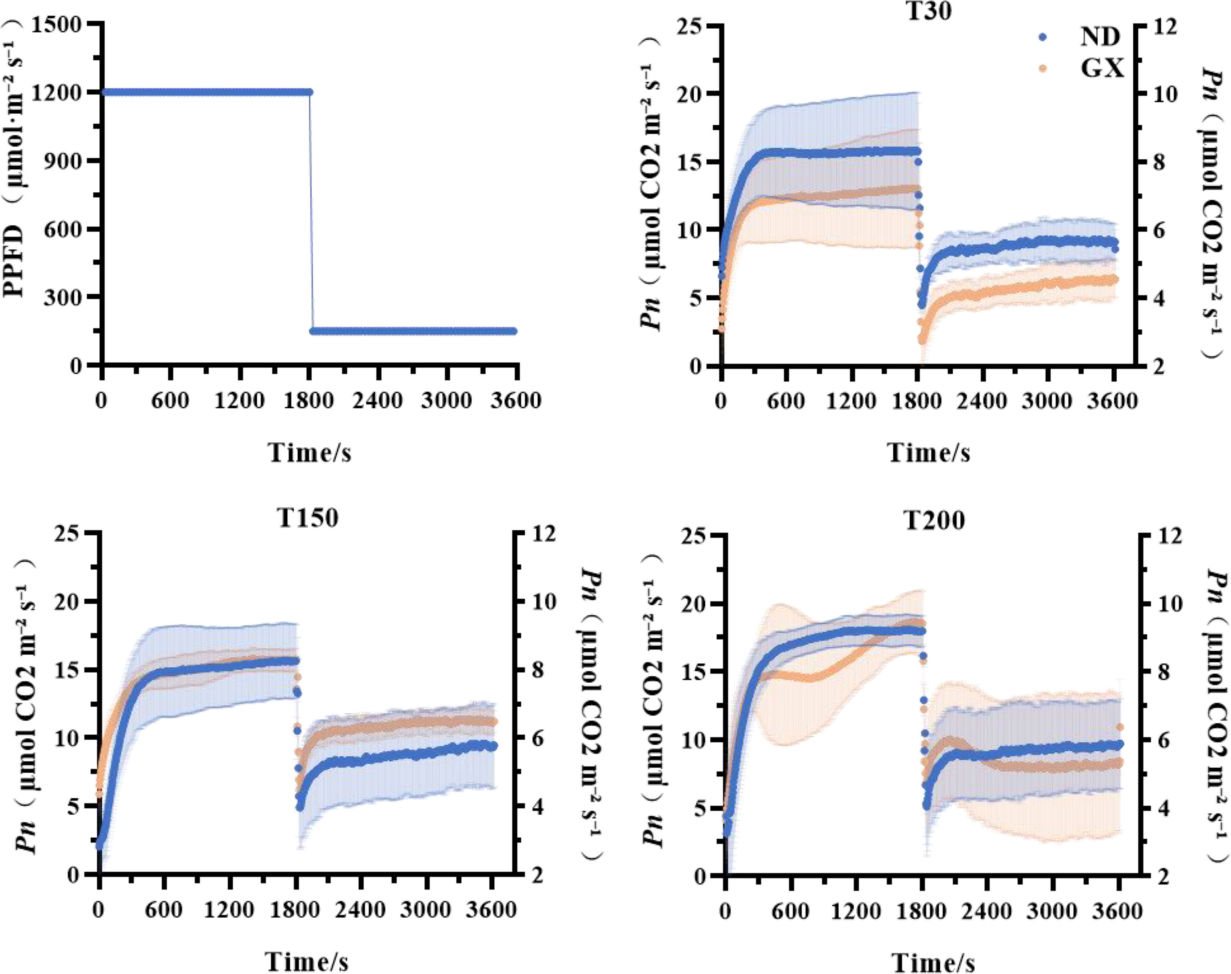
Effects of treatments with different durations of intense midday light on the slow photosynthetic response of soybeans.

Through photosynthetic induction analysis spanning low-high-low light transitions, we found no significant differences in rapid photosynthetic response capacity (time to 50% Pnmax) between the two soybean cultivars across treatments. While ND12 showed comparable photosynthetic acclimation capacity (time to 90% Pnmax) among treatments, GX7 exhibited significant enhancements of 206.53% and 152.69% under T150 and T200, respectively, compared to T30 (**Figure 3**; **Table 1**).

**Table 1:** The rapid response capacity (time to reach 50% of Pnmax) and full adaptation capacity (time to reach 90% of Pnmax) of photosynthesis of two soybean varieties under three light treatments.

Treatment		Time for net photosynthetic rate to rise to 50% of its maximum value(min)	Time for net photosynthetic rate to rise to 90% of its maximum value(min)
ND12	T30	2.2 ± 0.81a	9.37 ± 4.02a*
	T150	1.67 ± 0.77a	7.04 ± 3.93a
	T200	1.93 ± 0.72a	6.26 ± 0.84a
GX7	T30	1.97 ± 1.05a	18.58 ± 4.71a
	T150	1.43 ± 1.12a	6.06 ± 0.63b
	T200	1.5 ± 0.61a	7.35 ± 1.39b

Different letters denote significant differences (P < 0.05) among treatments, while * indicates significant differences (p < 0.05) between soybeans. Significance is the same as above.

**Figure 4:**
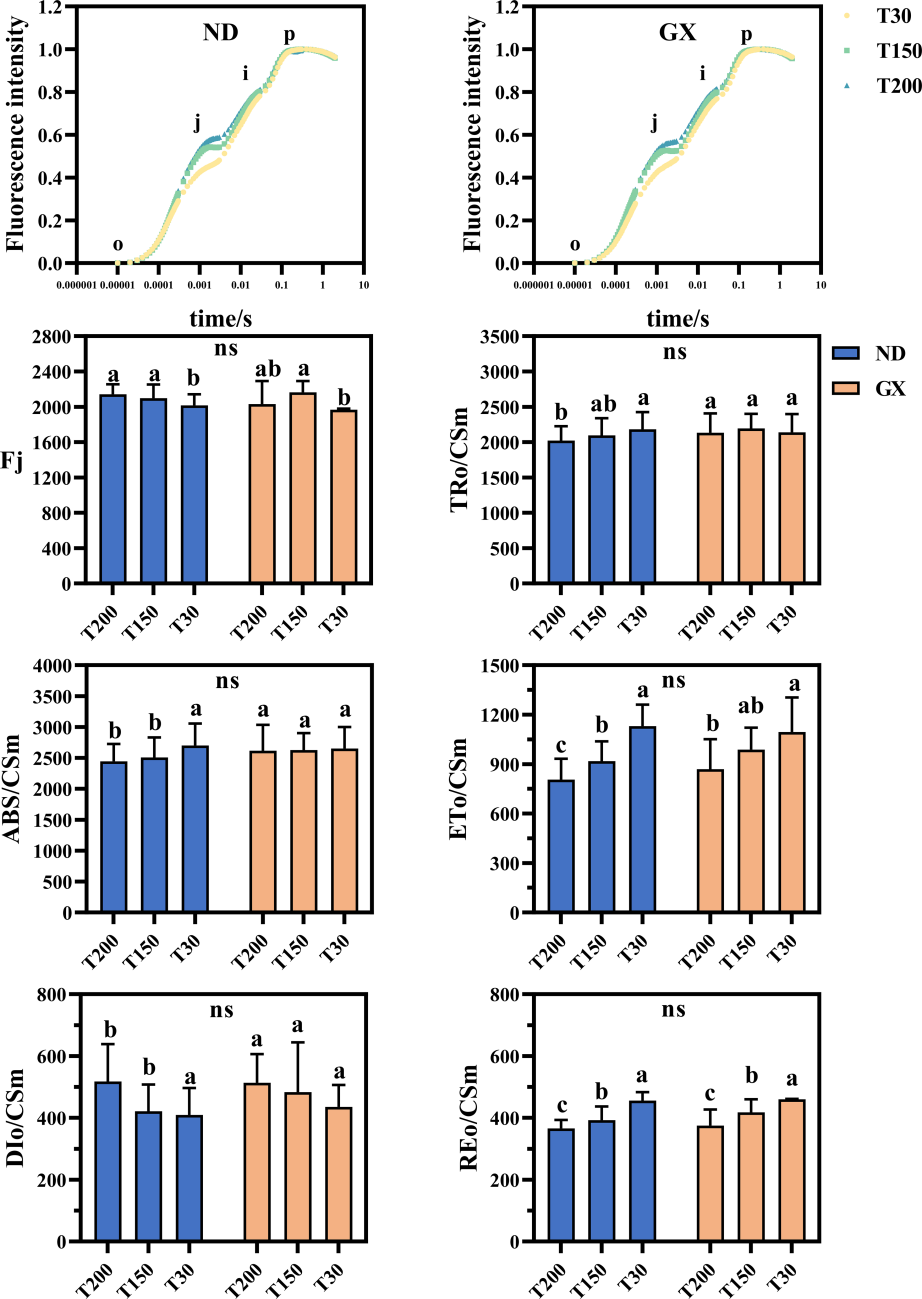
OJIP images and energy distribution parameters of the soybean photosynthetic system under different durations of high noon light treatments. Bars represent mean ± SEM (n = 10). Statistical differences (p < 0.05) were determined using one-way ANOVA with LSD *post hoc* test. Some significant differences may appear subtle due to small effect sizes but were statistically confirmed based on low within-group variability. “ns” indicates no significant difference among soybean treatments.

ND12 demonstrated 4.07% and 2.09% increases under T150 and T200 compared to T30, whereas GX7 showed a 10.05% enhancement exclusively under T150. Energy allocation patterns differed markedly between cultivars. ND12 displayed higher energy absorption per reaction center (ABS/Csm) and trapping efficiency (TRo/Csm) in T30 than in T150/T200 (**Figure 4**), while GX7 maintained comparable values across treatments. Under the T150 and T200 treatments, the electron transfer energy per reaction center (ETo/Csm) of both ND12 and GX7 decreased significantly, while the dissipated heat energy per reaction center (DIo/Csm) increased. The longer the duration of intense midday light, the lower the reduction energy per reaction center (REo/Csm) of ND12 and GX7. There were no significant differences in this regard between the two varieties.

### 3.3 Effects of the duration of intense midday light on the activity of the photosystem and electron transfer in soybeans

**Figure 5:**
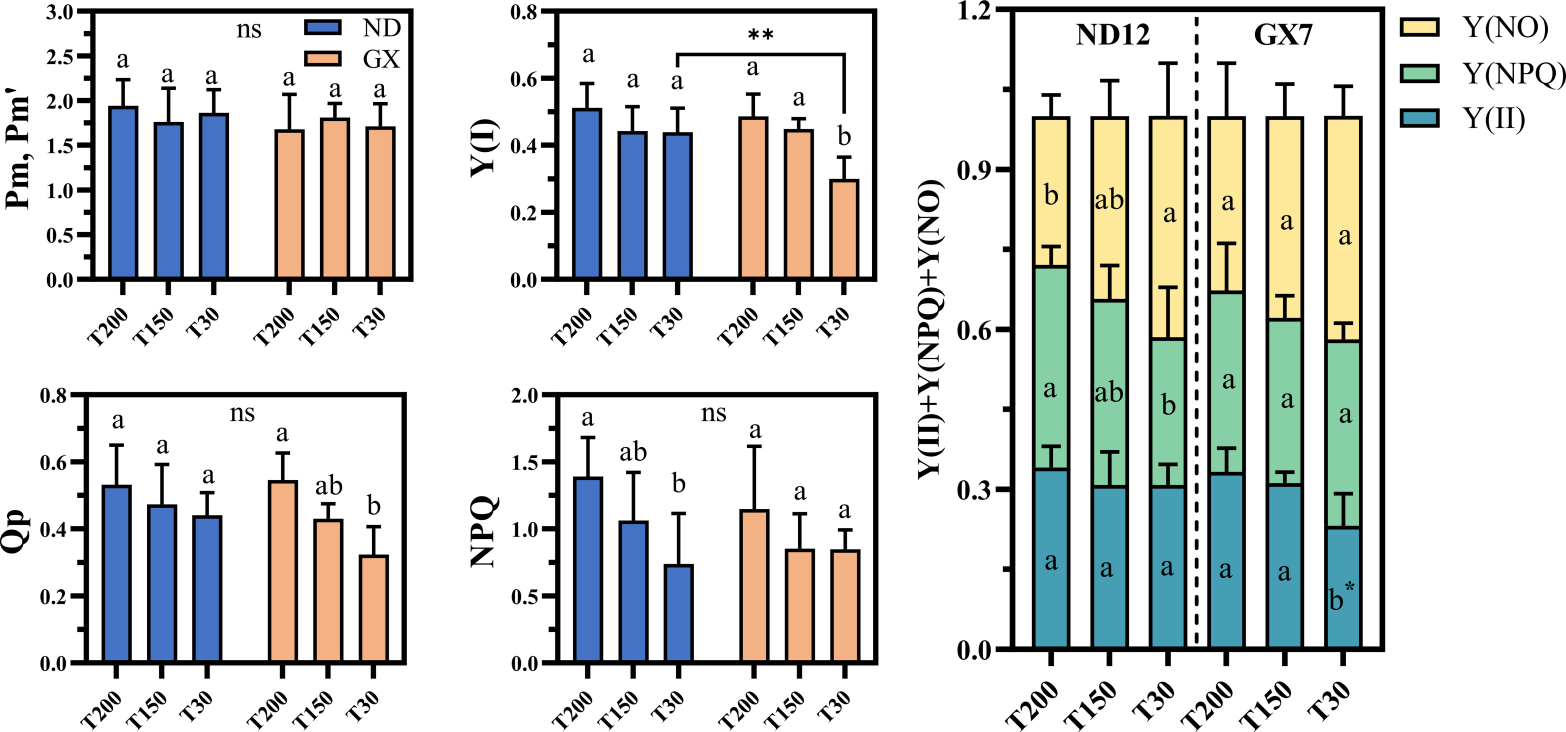
Effects of simulated light on the photosynthetic system of soybean leaves. Different lowercase letters indicate significant differences among light treatments within the same cultivar (p < 0.05, LSD). Asterisks (*) denote significant differences between cultivars under the same light treatment (**p < 0.01). “ns” indicates no significant difference among soybean treatments.

Both soybean cultivars exhibited common photoprotective responses to prolonged midday high-light exposure, including a significant increase in regulated energy dissipation [Y(NPQ)] and a concurrent decline in non-regulatory dissipation [Y(NO)] (**Figure 5**). These adjustments occurred alongside largely stable quantum yields of PSII and PSI [Y(II), Y(I)] and photochemical quenching (qP) across treatments, indicating a conserved baseline capacity to regulate excess excitation energy under variable light durations(SI.fig1). Despite these similarities, cultivar-specific differences emerged, particularly under short-term high-light conditions (T30). GX7 showed significant reductions in Y(II), Y(I), and qP under T30 compared with T200 (30.25%, 38.33%, and 40.50% decreases, respectively; P < 0.05), whereas ND12 maintained higher overall photosystem efficiency, with combined PSII–PSI quantum yields 22.6% greater than GX7 under T30.

**Figure 6:**
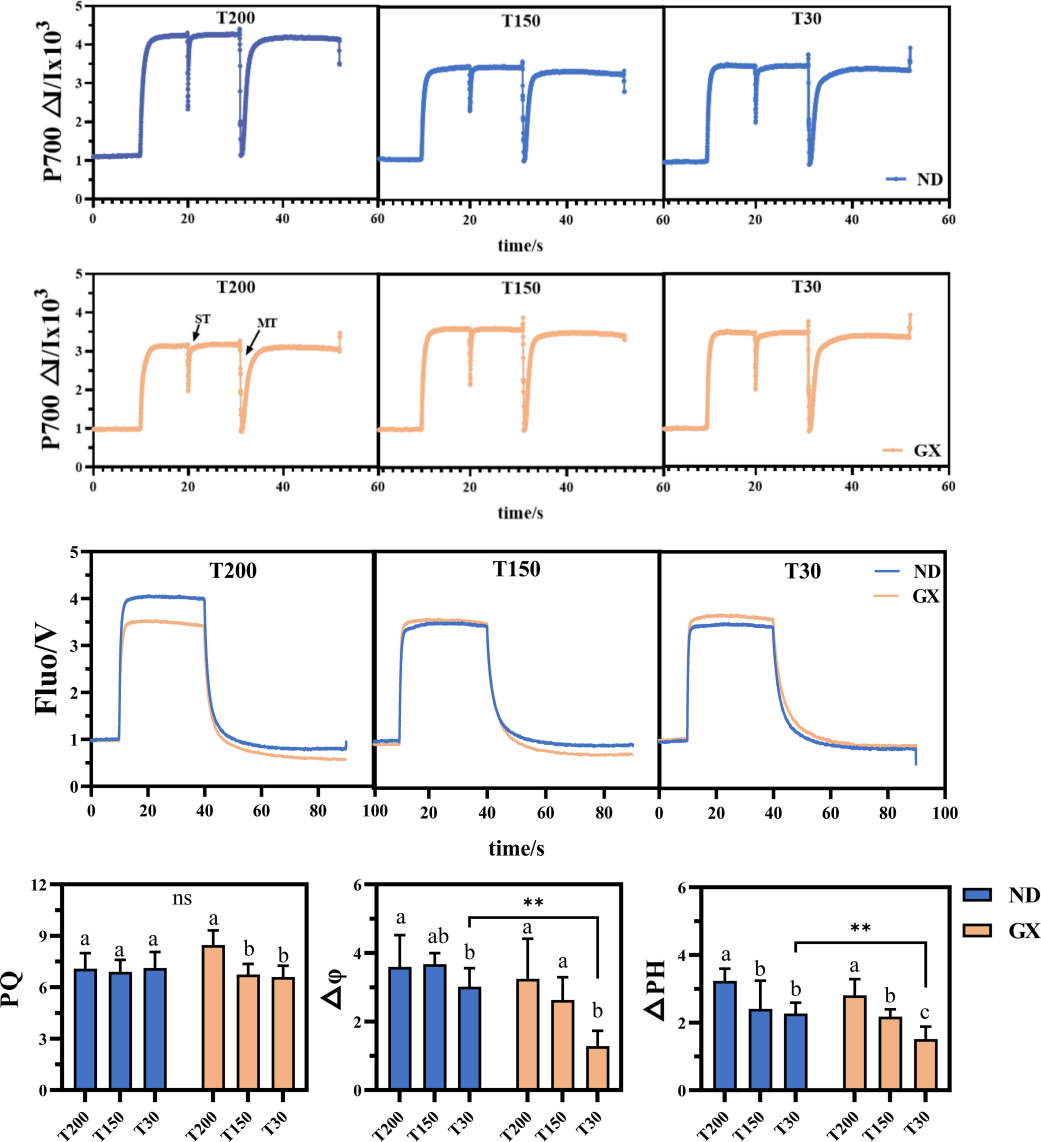
Effects of simulated light on the kinetics of the plastoquinone (PQ) pool, the dark recovery of P700 and the parameters of thylakoid energization, namely the membrane potential ΔΨ and the proton gradient ΔpH. These are expressed in normalized arbitrary units and do not correspond to absolute pH values in the lumen. ** denotes highly significant ones (p < 0.01).

Electron transport dynamics further distinguished the two genotypes (**Supplementary Figure 1**). ND12 displayed stable ETR(II) and ETR(I) across treatments, while GX7 exhibited pronounced increases in both parameters under T200 and T150, by 41.46% and 31.99%, respectively, relative to T30. CEF activity in GX7 increased substantially under prolonged high-light (by 60.47%), as did the CEF/Y(II) ratio (+40.20%), contrasting with the minimal variation observed in ND12. Analysis of PSI donor-side limitation [Y(ND)] revealed higher stress in GX7 under T30, with a 46.46% increase relative to ND12. Moreover, plastoquinone (PQ) pool capacity in GX7 increased by 21.96% from T30 to T200, whereas ND12 maintained consistent PQ redox status across treatments(**Figure 6**). The kinetics of P700 dark reversion accelerated progressively with longer high-light exposure in GX7; under T30, however, ND12 exhibited a 38.7% faster reversion rate, indicative of more efficient electron turnover.

Both cultivars responded to extended high-light exposure with elevated thylakoid energization (**Figure 6**). Prolonged treatments increased ΔΨ and ΔpH by 34.8% and 27.3%, respectively, but ND12 consistently showed higher energization under T30, with ΔΨ and ΔpH values 52.4% and 47.1% greater than GX7 (P < 0.01). Together, these results demonstrate a core set of photoprotective responses shared between genotypes, while highlighting contrasting strategies in energy partitioning and electron transport regulation under varying durations of intense midday light.

### 3.4 Effects of midday light duration on photosynthetic induction dynamics under fluctuating light conditions

**Figure 7:**
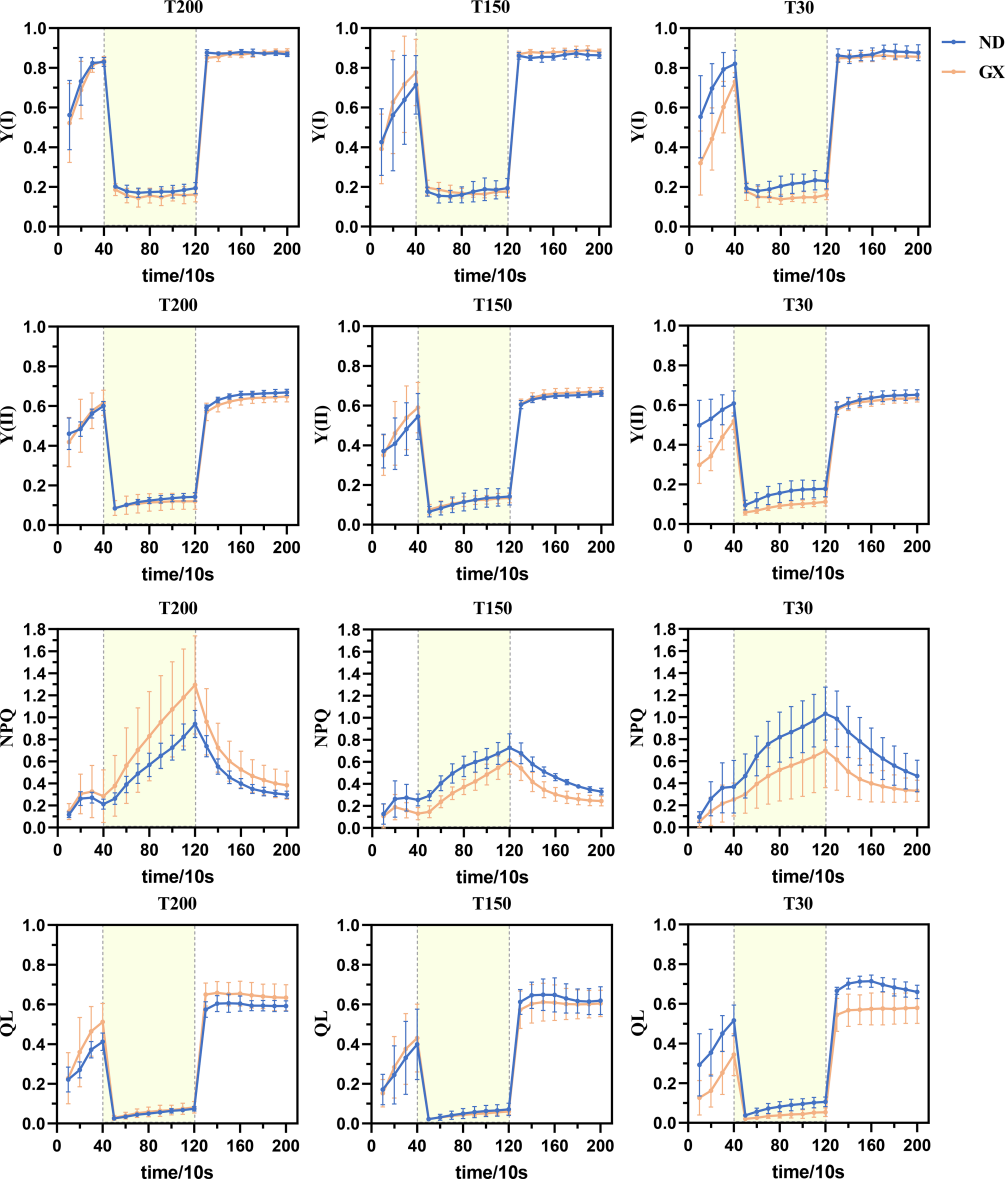
Under the alternating action of fluctuating light between 150-1200-150 μmol·m^−2^·s^−1^, changes in the parameters of photosystem I (PSI) and photosystem II (PSII) in soybean leaves were observed. These parameters include Y(I), the quantum yield of PSI photochemistry; Y(II), the quantum yield of PSII photochemistry; NPQ, non-photochemical quenching; and qL, the photochemical quenching coefficient based on the “lake model” of PSII. The data are presented as the mean ± standard deviation (n = 10). Y(ND), the limitation on the donor side of photosystem I; Y(NA), the degree of PSI over-reduction; Y(NO), the quantum yield of non-regulated energy dissipation in PSII;and qP, the photochemical quenching coefficient based on the “puddle model” of PSII (**Supplementary Figure 2**).

The photosynthetic induction responses of both soybean cultivars to low-high-low light transitions revealed significant treatment-dependent patterns (**Figure 7**). Under T200 and T150 treatments, ND12 and GX7 exhibited comparable kinetics in Y(I) and Y(II) fluctuations, characterized by initial increases during low light, subsequent decreases under high light, and recovery upon return to low light. However, under T30 treatment, GX7 showed significantly lower Y(I) and Y(II) increases than ND12 during the initial low-light phase (P<0.05). Notably, upon sudden light intensification, GX7 demonstrated dramatic reductions in Y(I) and Y(II) to 0.2 and 0.06, respectively, while ND12 maintained significantly higher values (P<0.01). PSI donor-side limitation analysis through Y(ND) revealed that all treatments showed gradual increases during low light, rapid elevation to 0.7 under high light, and sharp declines to approximately 0.1 upon light reduction, with GX7 under T30 exhibiting significantly higher Y(ND) than ND12 during high-light exposure.

Dark-adapted initial Y(NA) values (at 10s) followed the pattern T30 > T150 > T200, with GX7 under T30 maintaining significantly higher Y(NA) than ND12 throughout the light transitions. Following light intensification, Y(NA) peaked around 50s before gradually decreasing to 0.1, with subsequent slow declines during the final low-light phase. Prolonged midday illumination significantly enhanced GX7’s NPQ capacity while reducing its Y(NO), as evidenced by ND12’s consistently lower Y(NO) (non-regulated energy dissipation at PSII) and higher NPQ (regulated energy dissipation) across all light phases under T30 (P<0.05). Furthermore, midday light duration treatments improved GX7’s photochemical quenching capacity (QP and QL), though ND12 maintained superior QP and QL values than GX7 under T30 throughout the light transitions.

## 4 Discussion

### 4.1 Photoprotective coordination and electron transport adjustments under prolonged midday irradiance

Prolonged exposure to intense midday light acts as a crucial environmental signal driving convergent photoprotective responses in soybean. Under treatments with extended midday irradiance duration (row spacings corresponding to T150–T200), the two cultivars examined, consistently exhibited enhanced regulated energy dissipation, as reflected by significant increases in DIo/CSm (+18.7–22.3%) (**Figure 4**). This conserved adjustment highlights the central role of dynamic non-photochemical quenching (NPQ) in safeguarding the photosynthetic apparatus under prolonged high-light conditions, in line with previous observations across diverse species (Goss and Lepetit, 2015; Lazzarin et al., 2024). The robust induction of NPQ suggests that soybean cultivars share a common regulatory framework aimed at rapidly dissipating excess excitation energy and minimizing photoinhibition during midday peaks (Yang et al., 2015; Liu and Yang, 2024).

In addition to enhanced energy dissipation, both ND12 and GX7 displayed coordinated modifications in electron transport processes, indicative of regulation of the proton motive force (pmf) and cyclic electron flow (CEF) (Takahashi and Badger, 2011; Huang et al., 2019). These adjustments likely optimize ATP/NADPH balance and maintain thylakoid membrane stability under sustained light stress. Such photoprotective coordination reflects a generalized adaptive strategy that enhances resilience to temporally heterogeneous irradiance, particularly relevant for improving light capture and use efficiency in intercropping systems.

Despite these shared responses, cultivar-specific differences were observed in the modulation of electron transport. ND12 maintained a more stable ETR(II) and exhibited faster ΔpH formation under prolonged light, suggesting a constitutive activation of protective pathways advantageous for environments dominated by fluctuating light, such as shaded understories. In contrast, GX7 showed a marked increase in CEF with prolonged light exposure, indicative of an inducible photoprotective strategy that may confer benefits under more predictable light regimes. While these differences underline the existence of genotype-specific nuances, the overall findings emphasize a broadly conserved photoprotective framework among soybean cultivars in response to midday irradiance (Dang et al., 2023; Wu et al., 2023).

### 4.2 Redox memory and photosynthetic niche adaptation under midday irradiance

The patterns of proton motive force (pmf) regulation observed under prolonged midday light suggest a shared adaptive mechanism among soybean cultivars, centered on the dynamic adjustment of thylakoid redox states (Ho et al., 2022). Both ND12 and GX7 exhibited flexible modulation of ΔpH and ΔΨ components in response to increasing light duration, indicating that fine-tuning the balance between electric potential and proton gradient is a critical strategy for optimizing photosynthetic efficiency under fluctuating irradiance. Such regulation is essential for maintaining ATP production and preventing over-reduction of the electron transport chain, thereby stabilizing photosystem performance during intense midday light a feature highly relevant to relay intercropping systems where light environments are temporally heterogeneous (Wang et al., 2022b).

In addition, both cultivars demonstrated the capacity to adjust electron transport dynamics, including mechanisms that stabilize PSI redox poise and mitigate donor-side limitations under high light stress (Wang et al., 2022a; Wang et al., 2022b). These findings underscore that dynamic control of electron flow and pmf partitioning constitutes a fundamental photoprotective response across soybean genotypes exposed to extended midday irradiance (Yamori, 2016).

Despite this shared regulatory framework, subtle differences were observed between cultivars. ND12 showed a greater reliance on ΔΨ formation relative to ΔpH, along with faster P700 re-reduction kinetics in darkness, suggesting a strategy that emphasizes rapid ATP synthase activation and cyclic electron flow to enhance recovery under transient shading (Tanaka et al., 2019). In contrast, GX7 exhibited an expansion of the plastoquinone (PQ) pool under prolonged light exposure, coupled with delayed relaxation of donor-side PSI limitation, indicative of a potential “electron capacitor” mechanism to buffer excess reducing equivalents during high-light phases. These cultivar-specific nuances reflect alternative adaptations to midday light stress, although the overarching photoprotective strategies remain largely conserved.

### 4.3 Integrating photoprotective plasticity with agronomic design

These findings demonstrate that the duration of midday high-light exposure acts as a key driver modulating photoprotective plasticity in soybean, underscoring the critical role of temporal light dynamics beyond traditional intensity-focused frameworks (Ho et al., 2022). Both ND12 and GX7 exhibited dynamic adjustments in energy dissipation and electron transport processes, indicating a common capacity among soybean cultivars to optimize photoprotective responses according to the temporal characteristics of the light environment. Such flexibility has important implications for designing intercropping systems that account for not only light quantity but also its temporal distribution.

Although shared photoprotective strategies were evident, subtle cultivar-specific differences emerged. ND12 predominantly utilized rapid ΔpH-mediated NPQ induction, while GX7 favored CEF-dependent electron flow adjustment under prolonged illumination (SI.fig1), providing mechanistic insights into genotype-specific light adaptation. The observed expansion of the plastoquinone pool in GX7 under T200 conditions (21.96%) further suggests a potential role for redox-mediated retrograde signaling in fine-tuning duration-dependent photoprotection. Future studies incorporating time-resolved transcriptomic analyses and multi-environment field trials will be essential to unravel the molecular underpinnings of these responses and to validate their agronomic relevance under diverse cropping conditions.

## 5 Conclusions

This study highlights the pivotal role of midday high-light exposure in regulating photoprotective mechanisms and electron transport adjustment in soybean cultivars. Under controlled conditions simulating prolonged midday irradiance, both ND12 and GX7 exhibited dynamic photoprotective responses, including modulation of cyclic electron flow (CEF) and proton motive force components, reflecting a conserved physiological plasticity essential for maintaining photosynthetic efficiency under fluctuating light environments.

Despite these shared adaptive features, cultivar-specific differences were evident. ND12 maintained more stable electron transport via sustained ΔpH and CEF activation and sustained ΔpH generation, supporting a greater capacity for low-light acclimation. In contrast, GX7 showed dual limitations in electron transport under reduced light, associated with lower CEF activity and less coordinated photosystem regulation. These results suggest that while both cultivars possess intrinsic mechanisms to cope with midday high-light exposure, their differential regulatory strategies may influence their performance under varying canopy light conditions. Future Light intensity moderates restudies under field conditions are warranted to evaluate the agronomic relevance of these physiological traits in diversified cropping systems.

## Data Availability

The raw data supporting the conclusions of this article will be made available by the authors, without undue reservation.
