# Supplementary material for: The adaptability of soybean photosynthesis to midday high-light duration through CEF-NPQ coupling regulation
Source: Front Plant Sci. 2025 Sep 9;16:1648079. doi: 10.3389/fpls.2025.1648079 (PMC12454400; doi:10.3389/fpls.2025.1648079)
Supplement: Supplementary file 1 [file DataSheet1.docx]

| Parameter | Symbol | Formula | Description |
| --- | --- | --- | --- |
| Quantum yield of PSI photochemistry | Y(I) | $\frac{{P^{'}}_{m}-P}{P_{m}}$ | Reflects the open ratio of the PSI reaction center |
| Oxidation ratio of P700 | Y(ND) | $\frac{P}{P_{m}}$ | Characterizes the electron transfer capacity of the PSI donor side |
| Extent of PSI over-reduction | Y(NA) | $\frac{P_{m}-{P^{'}}_{m}}{P_{m}}$ | Related to cyclic electron flow and photoprotection mechanism |
| Quantum yield of PSII photochemistry | Y(II) | $\frac{{F^{'}}_{m}-F_{s}}{{F'}_{m}}$ | Reflects the energy conversion efficiency of the open reaction center |
| Quantum yield of non-regulated energy dissipation | Y(NO) | $\frac{F_{s}}{F_{m}}$ | Related to the risk of photodamage |
| Non-photochemical quenching | NPQ | $\frac{F_{m}-{F'}_{m}}{{F'}_{m}}$ | Reflects the ability to dissipate heat |
| Photochemical quenching (puddle model) | qP | $\frac{{F^{'}}_{m}-F_{s}}{{F'}_{m}-{F'}_{o}}$ | Reflects the open ratio of the PSII reaction center |
| Photochemical quenching (lake model) | qL | $qP\times\frac{{F'}_{O}}{F_{S}}$ | Correction of energy transfer between PSII units |
| Max. efficiency of open PSII centers | $\frac{{F^{'}}_{v}}{{F'}_{m}}$ | $\frac{{F^{'}}_{m}-{F'}_{o}}{{F'}_{m}}$ | Maximum photochemical efficiency of open PSII centers under illumination |
| Plastoquinone pool size | PQ_size_ | $\frac{{MT}_{area}}{{ST}_{area}}$ | Reflects the redox state of the downstream electron transport chain |

**Table 1** Definitions and formulas of photochemical parameters for PSI, PSII, and plastoquinone pool

Note: Abbreviations and physiological significance of each photochemical parameter.


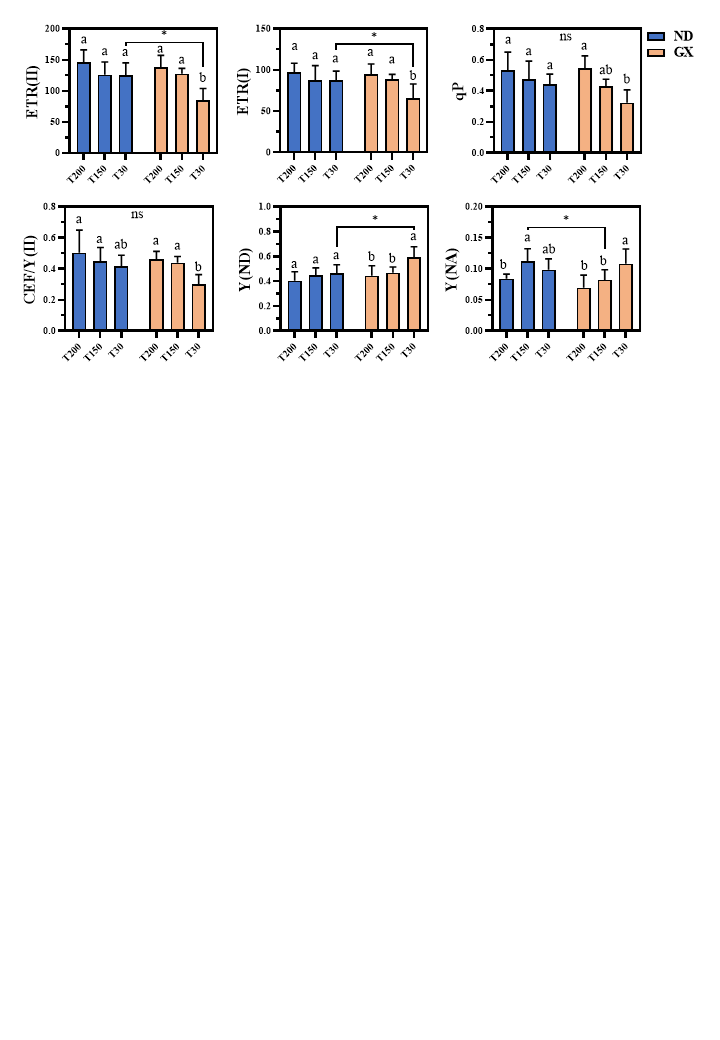


**SI.Figure1** Effects of simulated light on the electron transfer rates of soybean leaves

Note: Different lowercase letters indicate significant differences among light treatments within the same cultivar (p < 0.05, LSD). Asterisks (*) denote significant differences between cultivars under the same light treatment (*p < 0.05, **p < 0.01).


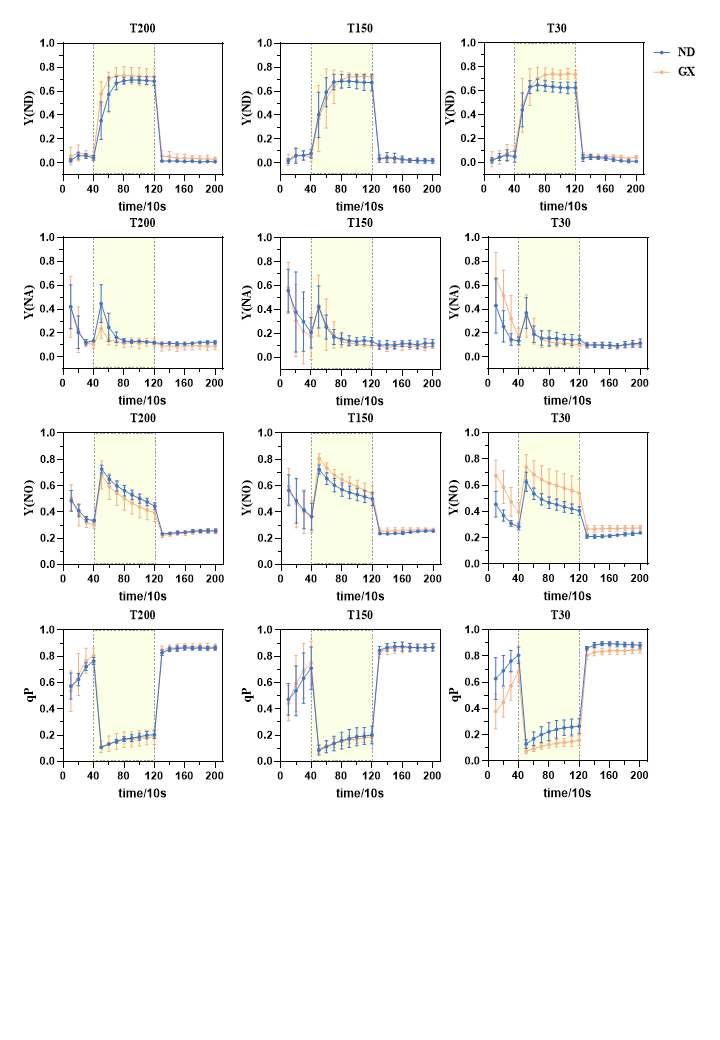


**SI.Figure2** Under the alternating action of fluctuating light between 150-1200-150 μmol photons·m⁻²·s⁻¹, changes in the parameters of photosystem I (PSI) and photosystem II (PSII) in soybean leaves were observed. Y(NA), the degree of PSI over-reduction; Y(NO), the quantum yield of non-regulated energy dissipation in PSII;and qP, the photochemical quenching coefficient based on the "puddle model" of PSII.

**Table 2** Pigment contents in leaves of two soybean cultivars under different light treatments

| Parameter | Cultivar | T200 (min) | T150 (min) | T30 (min) |
| --- | --- | --- | --- | --- |
| Chl a | ND12 | 12.74 ± 2.60 | 8.82 ± 1.81 | 8.67 ± 2.31 |
|  | GX7 | 12.10 ± 1.04 | 12.07 ± 1.93 | 7.59 ± 1.59 |
| Chl b | ND12 | 4.53 ± 0.90 | 3.19 ± 0.69 | 1.78 ± 0.79 |
|  | GX7 | 3.75 ± 0.78 | 4.45 ± 0.48 | 2.04 ± 0.73 |
| Carotenoid | ND12 | 2.58 ± 0.74 | 1.93 ± 0.33 | 1.69 ± 0.44 |
|  | GX7 | 2.38 ± 0.62 | 2.55 ± 0.33 | 1.29 ± 0.65 |
| Chl a/b | ND12 | 2.78 ± 0.08 | 2.77 ± 0.12 | 4.27 ± 0.18 |
|  | GX7 | 2.82 ± 0.10 | 3.01 ± 0.46 | 4.19 ± 0.88 |
